# Lower spinal levels and male sex are associated with greater epidural blood patch volume in spontaneous intracranial hypotension

**DOI:** 10.1186/s10194-025-02015-1

**Published:** 2025-04-14

**Authors:** Woo-Seok Ha, JaeWook Jeong, Seungwon Song, Jungyon Yum, Soomi Cho, Hee Jung Kim, Min Kyung Chu

**Affiliations:** 1 Department of Neurology, Yonsei University College of Medicine, 50-1 Yonsei-ro, Seodaemun-gu, Seoul, Republic of Korea; 2 Department of Anesthesiology and Pain Medicine, Anesthesia and Pain Research Institute, Yonsei University College of Medicine, 50-1 Yonsei-ro, Seodaemun-gu, Seoul, Republic of Korea

**Keywords:** Epidural blood patch, Spontaneous intracranial hypotension, Intracranial hypotension, Cerebrospinal fluid leak, Headache disorders

## Abstract

**Background:**

The epidural blood patch (EBP) is the treatment of choice for spontaneous intracranial hypotension (SIH). Studies have shown that targeted EBP is more effective than blind EBP. Additionally, a greater volume of injected blood during EBP has been associated with better therapeutic outcomes. However, symptoms such as back pain often prevent achieving the desired blood volume. This study aimed to analyse factors influencing the tolerable EBP volume, including structural, clinical, and psychological factors.

**Methods:**

This retrospective study included patients diagnosed with SIH who underwent single-level EBP at a tertiary care centre from 2019 to 2024. Data collected encompassed target levels, cross-sectional area, types of EBP, demographics, imaging findings, maximum intensity of orthostatic headache, Headache Impact Test-6, psychological state (Generalized Anxiety Disorder-7 and Patient Health Questionnaire-9), and somatic symptom burden (Widespread Pain Index and Symptom Severity Scale). A linear mixed model (LMM) was used to investigate factors influencing the total injected blood volume, accounting for repeated EBP procedures per patient. Sensitivity analysis was performed to assess model robustness.

**Results:**

A total of 103 EBP procedures from 53 patients (62% female; mean age, 39.9 ± 11.1 years) were analysed. The results of the LMM revealed that lower spinal levels (beta = 0.306, *P* = 0.029) and male sex (beta = 4.347, *P* = 0.024) were significantly associated with higher tolerable EBP volumes. Psychological factors or somatic symptom burden did not have a significant impact on the injected blood volume. In the sensitivity analysis, the number of EBP procedures (beta = -0.804, *P* = 0.001) was also significantly associated with lower tolerable EBP volume.

**Conclusions:**

Lower spinal levels and male sex were associated with higher tolerable EBP volumes in patients with SIH. The trade-off between spinal level and tolerable EBP volume should be considered when developing targeted blood patch strategies and evaluating their efficacy.

**Graphical Abstract:**

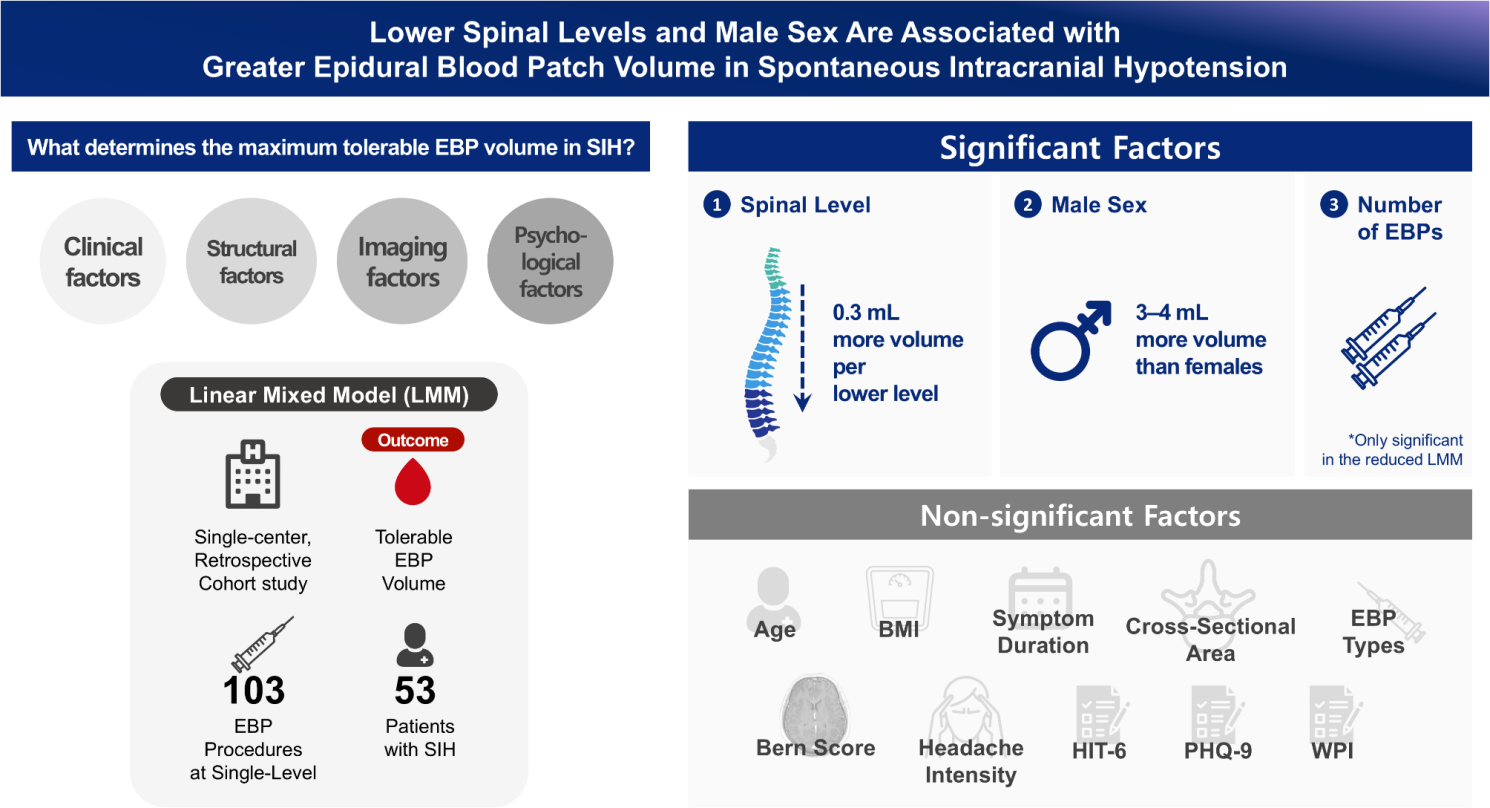

**Supplementary Information:**

The online version contains supplementary material available at 10.1186/s10194-025-02015-1.

## Background

Spontaneous intracranial hypotension (SIH) is a condition caused by spinal cerebrospinal fluid (CSF) leakage, with symptoms including orthostatic headache, nausea, posterior neck pain, tinnitus, and altered vision [1]. The epidural blood patch (EBP) is the mainstay treatment for SIH, as it directly addresses the underlying cause—spinal CSF leakage—by sealing the leak and restoring CSF pressure and volume, thereby effectively alleviating symptoms [2]. The response rate to a single EBP in SIH varies widely, ranging from 29 to 68% [3–6].

While differences in how responses are measured across studies contribute to this variability, a more fundamental reason lies in the heterogeneity of EBP techniques, including targeted vs. blind, single-level vs. multi-level, interlaminar vs. transforaminal methods, use of autologous blood only vs. fibrin mixed, and large-volume vs. small-volume injections [7].

Recent studies have reported that targeted EBP demonstrates a superior treatment response compared to blind EBP and that a larger injection volume is critical for efficacy [3, 4, 6, 8]. These findings will provide essential evidence in establishing optimal strategies for EBP in the management of SIH. However, the desired injection volume cannot always be achieved due to patient tolerability during the procedure [9]. Immediate side effects of EBP include not only back pain but also radiculopathy, nausea, and hypotension. Furthermore, rare but serious complications, such as permanent paraparesis or cauda equina syndrome, have been reported, leading to the recommendation to discontinue the injection if pain occurs during the procedure [10, 11]. Given these characteristics, which rely heavily on the patient’s subjective tolerance, EBP volume exhibits significant variability between individuals.

In this study, we aimed to identify the factors influencing tolerable EBP volume. These factors included structural and clinical elements, such as target levels and headache severity, as well as psychological aspects, such as anxiety and somatic symptom burden. Through this analysis, we sought to provide insights into the key considerations necessary for developing effective EBP strategies in clinical practice.

## Methods

### Participants

This study utilized retrospectively collected data from patients who met the inclusion criteria. The inclusion criteria for study participants were as follows: (1) adult patients diagnosed with SIH from 2019 to 2024 at our tertiary care centre; (2) patients who underwent a single-level EBP at our centre. The exclusion criteria included a lack of magnetic resonance imaging (MRI) or patients who had undergone previous spinal interventions prior to the onset of symptoms. The diagnosis of SIH was confirmed through imaging or low CSF pressure (defined as an opening pressure of < 60 mm CSF on a spinal tap), consistent with the International Classification of Headache Disorders criteria for SIH diagnosis [12].

Demographic data, including age, sex, height, weight, the date of symptom onset, and history of attempted epidural blood patch procedures prior to referral, were collected from all patients. During the first outpatient visit, patients completed and submitted the following questionnaires, either on mobile devices or on paper: maximum headache intensity (Numeric Rating Scale [NRS]), Headache Impact Test-6 (HIT-6) [13], Generalized Anxiety Disorder-7 (GAD-7) [14], Patient Health Questionnaire-9 (PHQ-9) [15], Widespread Pain Index (WPI), and Symptom Severity Scale (SSS) [16].

### Standard of care and selection of EBP level

**Fig. 1:**
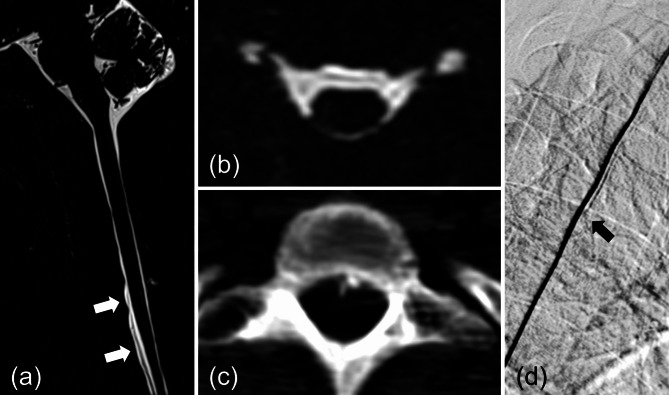
Imaging modalities used for patient evaluation and selection of EBP level. (**a**) Sagittal view of spine MRI with heavily T2-weighted fat-saturated imaging showing spinal longitudinal extradural fluid (SLEC). (**b**) Axial view of spine MRI showing ventral SLEC along with bilateral nerve root diverticula. (**c**) Noncontrast spine CT identifying the presence of discogenic microspurs. (**d**) Digital subtraction myelography (DSM) demonstrating a localised CSF leak at a mid-thoracic level

All patients underwent brain MRI with contrast, from which the Bern score was calculated [17]. Spine MRI, with or without heavily T2-weighted fat-saturated imaging, was utilised to identify the presence and spinal levels of spinal longitudinal extradural fluid (SLEC) and nerve root diverticula (Fig. 1a and b). SIH was radiologically diagnosed if the Bern score on brain MRI was ≥ 5 (brain-positive) or if SLEC was observed on spine MRI (spine-positive). In addition, at our institution, non-contrast whole spine computed tomography (CT) is included in the initial evaluation to assess the presence of discogenic microspurs (Fig. 1c).

To prevent terminological confusion, we have defined the types of EBP as follows: Targeted EBP refers to procedures performed at a leakage site identified through digital subtraction myelography (DSM) or dynamic CT myelography (CTM). Semi-targeted EBP refers to procedures performed at a presumed leakage site based on spinal imaging findings, such as the location of bony spurs, diverticula, or SLEC, all of which are highly associated with the leakage site [18]. Blind EBP refers to procedures performed at anatomical landmarks, regardless of the location of CSF leakage.

**Fig. 2:**
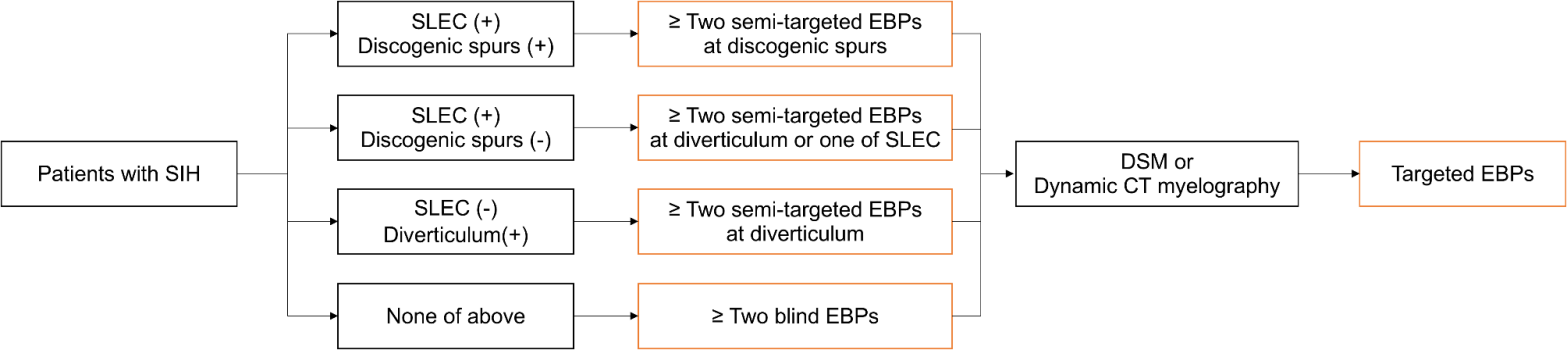
Decision tree for selecting the EBP level. EBP, epidural blood patch; SLEC, spinal longitudinal extradural fluid; DSM, digital subtraction myelography; SIH, spontaneous intracranial hypotension; CT, computed tomography

The level for EBP was determined based on the following flow (Fig. 2). (1) If a patient had SLEC along with discogenic microspurs, the semi-targeted EBP was performed at one of the levels of the discogenic microspurs. (2) If SLEC was present without a discogenic microspur, the semi-targeted EBP was performed at the level of a meningeal diverticulum or the level with the most prominent epidural collection. (3) If SLEC was absent but a meningeal diverticulum was present, the semi-targeted EBP was performed at the level of the diverticulum. (4) If none of the above criteria were met or spinal imaging was unavailable, a blind EBP was performed. Blind EBP was primarily targeted at the lumbar level. If additional EBP was required, the cervicothoracic junction or mid-thoracic level was selected at the physician’s discretion.

If the patient’s clinical symptoms did not completely resolve, repeated EBPs were performed at intervals of at least 2 weeks following an interdisciplinary discussion. If symptoms did not improve after two EBP procedures, DSM or dynamic CTM was performed (Fig. 1d). These exams were performed using a dynamic technique. When the CSF leakage level was successfully localised, a targeted EBP was performed at the identified site. As this standard was established in 2023, patients who were seen prior to 2023 underwent blind EBP procedures.

### Epidural blood patch technique

All EBP procedures were performed in an inpatient setting. EBP procedures were carried out either by HJK, an anesthesiologist with substantial clinical expertise or by other anesthesiologists under HJK’s guidance. All EBPs were performed at a single level using an interlaminar approach, utilising only autologous blood with no fibrin mixture. Patients were pre-educated about the potential occurrence of mild headache, neck or back pain, radicular pain, discomfort, or dyspnoea during the injection of contrast medium or blood. They were also informed that the maximal injecting volume was 20 mL and instructed to immediately inform the medical staff if they felt unable to continue with the injection, allowing for its prompt discontinuation.

Patients were placed in the prone position and prepared under sterile conditions. Local anaesthesia with 1% lidocaine was administered to the skin and soft tissue at the intended entry site. Using an anteroposterior fluoroscopic view, a 20-gauge, 10-cm Tuohy needle was advanced to the designated intervertebral space. Entry into the epidural space was confirmed using the loss of resistance technique. Once the needle was positioned, a lateral fluoroscopic view was employed to ensure accurate needle placement and to confirm appropriate epidural spread by administering 1–2 mL of contrast medium.

A skilled nurse inserted a peripheral intravenous line and collected 20 mL of autologous blood. The blood was slowly injected through the Tuohy needle until the patient reached their tolerance limit or a maximum volume of 20 mL was delivered.

Following the procedure, the total volume of blood injected, the patient’s symptoms, the pain intensity immediately after the procedure (at the injection site or any immediate discomfort, including radicular pain, assessed by the NRS), and their vital signs, including blood pressure and oxygen saturation, were recorded in the medical chart. Patients were required to remain on bed rest for at least 6 h after the procedure. During this period, except for bathroom use and meals, they were instructed to maintain either a prone or supine position.

### Imaging analysis

The Bern score from initial brain MRI was collected. Spine MRI was used to identify the levels of SLEC, with separate assessments conducted for ventral and dorsal SLEC.

**Fig. 3:**
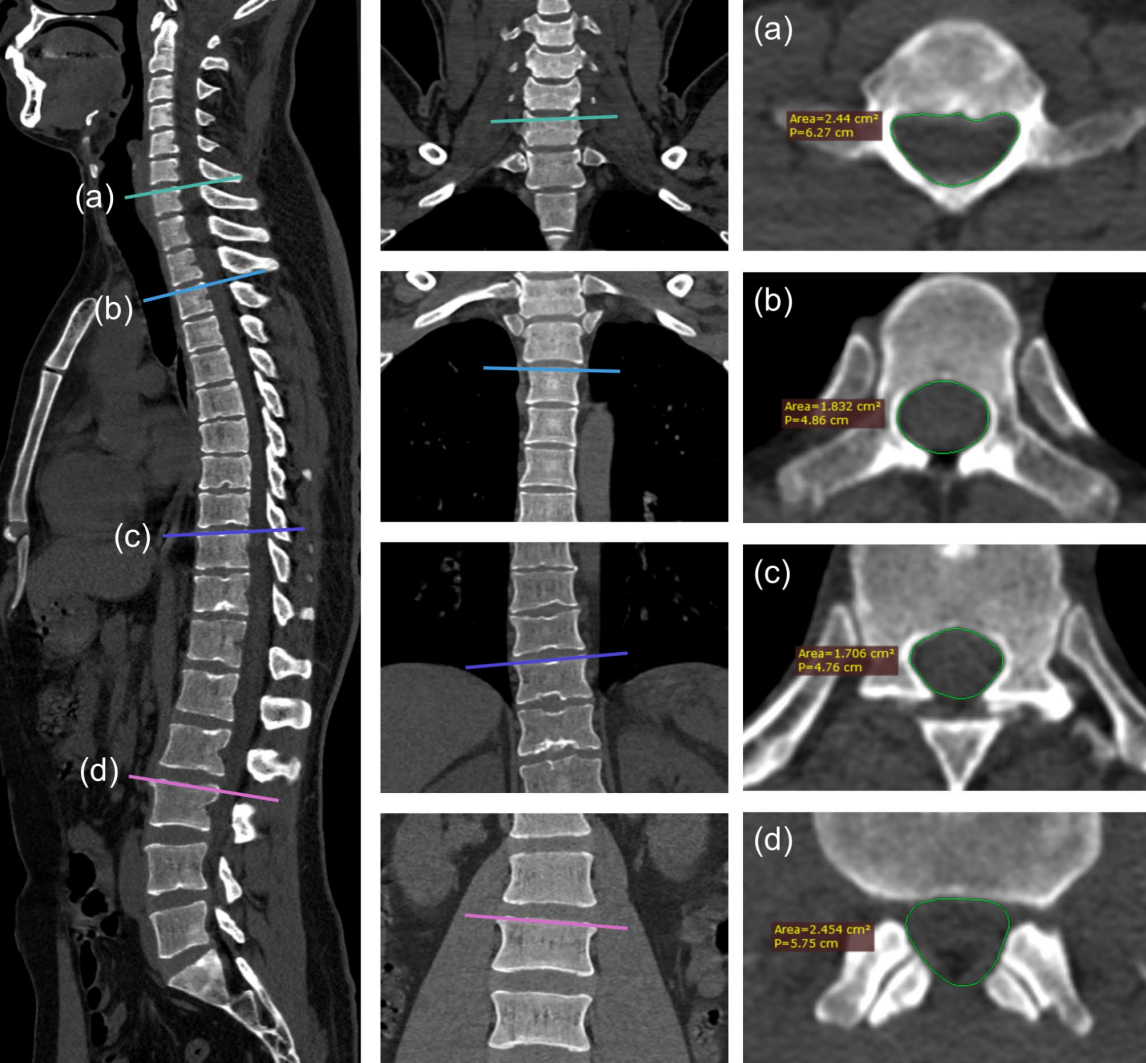
Measurement of spinal canal cross-sectional area at different levels. The left and middle panels show sagittal and coronal views, with colored lines indicating the measurement planes (**a**-**d**). The right panel presents the corresponding axial CT slices, where the spinal canal cross-sectional area was manually delineated (green outline). (**a**) C6-7 level. (**b**) T2-3 level. (**c**) T9-10 level. (**d**) L2-3 level

The cross-sectional area of the spinal canal was measured at the target EBP level using whole-spine CT with a 0.6-mm slice thickness. The CT images were reconstructed into a 3D dataset, and cross-sectional area was measured at each intervertebral level, specifically at the upper endplate of the inferior vertebra (Fig. 3). The measurement plane was aligned with the vertebra in the X, Y, and Z axes using the 3D-reconstructed CT images to ensure standardization. Manual measurements were performed by a single investigator (WSH).

### Statistical analysis

We conducted a linear mixed model (LMM) analysis to investigate the factors influencing the total injected blood volume during EBP, accounting for repeated measurements within individuals who underwent multiple EBP procedures. The total injected blood volume was set as the dependent variable. Independent variables included EBP-related factors (e.g., target level, days since symptom onset, EBP type, the number of EBP procedures, cross-sectional area of the target spinal canal, and whether the EBP level was within the extension of the SLEC), demographic data (age, sex, and body mass index [BMI]), imaging findings (Bern score and the presence of dorsal SLEC), maximum headache intensity (NRS), headache-related impact (HIT-6), psychological state (GAD-7 and PHQ-9), and somatic symptom burden (WPI and SSS). For analytical purposes, the target level was assigned numeric values sequentially, with C1-C2 as 1, T3-T4 as 10, and L5-S1 as 24.

Multicollinearity was evaluated using correlation coefficients (r), and variables exhibiting high correlations (|r| > 0.6) were further assessed. Among the correlated variables, the one resulting in a model with lower Akaike Information Criterion and Bayesian Information Criterion values was retained. The LMM was estimated using restricted maximum likelihood and included random intercepts to account for inter-individual variability, treating each patient as a random effect. Missing values were handled using maximum likelihood estimation inherent to LMM, which does not require listwise deletion and permits the inclusion of all available data. Fixed effects were included to evaluate the impact of the independent variables on the dependent variable.

To assess the robustness of the findings, a sensitivity analysis was performed. First, each independent variable was analysed using univariable LMM. Variables with P-value < 0.10 in univariable analyses were then included in a reduced multivariable LMM to identify the most influential factors while minimizing potential overfitting. In this reduced model, multicollinearity was reassessed.

Statistical significance was defined as a two-sided P-value of < 0.05. All analyses were conducted using SPSS Statistics for Windows, version 28.0 (IBM, Armonk, NY, USA).

## Results

A total of 103 EBP procedures from 53 patients with SIH were included (Table 1). All patients were Korean except for one Caucasian individual. Among the patients, 62% were female, and the mean age was 39.9 years. The median Bern score was 5 (interquartile range [IQR] 3–6), and 87% of patients exhibited SLEC on myelography. The median number of days since symptom onset was 26 (IQR 15–71). Questionnaire scores were available in 42 out of 53 patients and demonstrated significant variations in impact of headache (HIT-6: 65 [IQR 58–73]), psychological state (GAD-7: 7 [IQR 3–12], PHQ-9: 6 [IQR 3–12]), and somatic symptom burden (WPI: 2 [IQR 1–4], SSS: 5 [IQR 3–7]).

**Table 1:** Demographic and clinical characteristics of patients and epidural blood patches

	Patients with SIH (*n* = 53)
Female (%)	33 (62%)
Age, mean (SD)	39.9 (11.1)
BMI, mean (SD) (kg/m^2^)	23.6 (4.0)
Bern score, median (IQR)	5 (3–6)
Days after onset, median (IQR)	26 (15–71)
Attempted EBPs before referral, median (IQR)	0 (0–2)
Presence of SLEC on myelography (%)	46 (87%)
Ventral SLEC (%)	38 (83%)
Dorsal SLEC (%)	25 (54%)
Maximum headache intensity*, median (IQR) (NRS)	7 (6–8)
HIT-6*, median (IQR)	65 (58–73)
GAD-7*, median (IQR)	7 (3–12)
PHQ-9*, median (IQR)	6 (3–12)
WPI*, median (IQR)	2 (1–4)
SSS*, median (IQR)	5 (3–7)
Total EBPs per patient, median (IQR)	3 (2–4)
	Total EBPs (*n* = 103)
Injected blood volume, median (IQR) (mL)	13 (9–17)
Target spinal levels	
Cervical (%)	11 (11%)
Thoracic (%)	79 (77%)
Lumbar (%)	13 (13%)
Cross sectional area of target spinal canal (mm^2^)	
Cervical, mean (SD)	188.7 (13.2)
Thoracic, mean (SD)	159.3 (30.7)
Lumbar, mean (SD)	282.0 (74.9)
Target selection	
Blind EBPs	33 (32%)
Semi-targeted EBPs	56 (54%)
Targeted EBPs	14 (14%)

* Questionnaire scores were obtained from 42 patients

SD: standard deviation, BMI: body mass index, IQR: interquartile range, EBP: epidural blood patch, SLEC: spinal longitudinal extradural collection, DSM: digital subtraction myelography, CTM: computed tomography myelography, HIT-6: Headache Impact Test-6, NRS: Numeric Rating Scale, GAD-7: Generalized Anxiety Disorder-7, PHQ-9: Patient Health Questionnaire-9, WPI: Widespread Pain Index, SSS: Symptom Severity Scale

A median of three EBP procedures was performed per patient, with a median EBP volume of 13 mL (IQR 9–17). Among the 53 patients, 10 (19%) underwent only one EBP, 14 (26%) received two EBPs, 13 (25%) had three EBPs, and 16 (30%) required four or more EBPs. There was no difference in mean EBP volume at first (14.4 mL, SD 4.5), second (13.7 mL, SD 4.5), and third (12.5 mL, SD 4.7) EBPs in analysis of variance (*P* = 0.144). The median pain severity assessed by the NRS immediately after the procedure was 3 (IQR 2–4). The thoracic level was the most common targeted (79 procedures, 77%), followed by the lumbar (13 procedures, 13%) and cervical levels (11 procedures, 11%). The mean cross-sectional area of the spinal canal varied by spinal level, with cervical levels measuring 188.7 mm² (SD 13.2), thoracic levels 159.3 mm² (SD 30.7), and lumbar levels 282.0 mm² (SD 74.9). Among the procedures, 14% were targeted EBPs performed after positive findings on DSM or dynamic CTM, while 32% were blind EBPs.

**Fig. 4:**
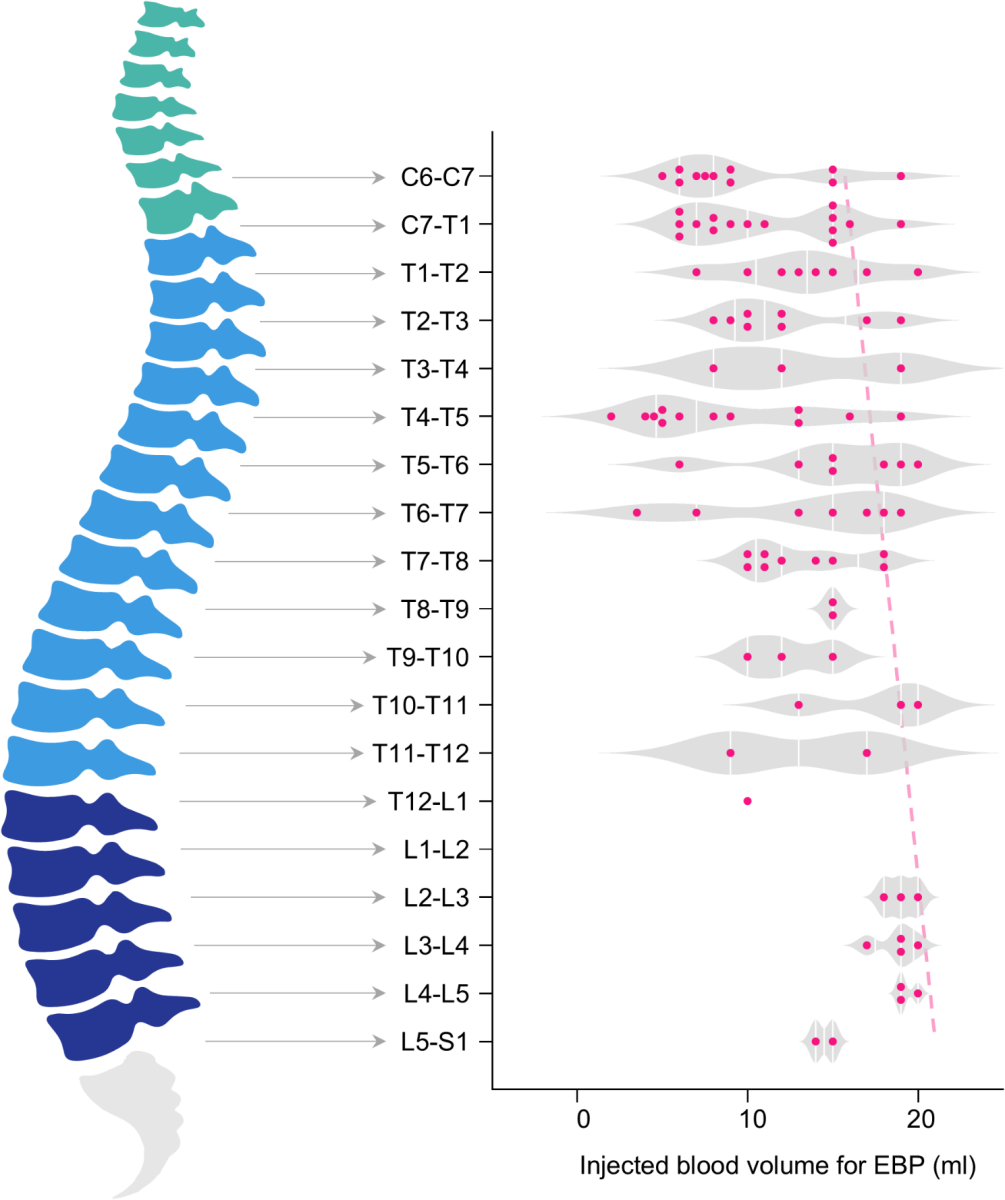
Violin plots of injected blood volume by spinal levels. Violin plots demonstrating the distribution of injected blood volumes stratified by spinal levels. The lower spinal levels show higher tolerable injected blood volumes, consistent with the findings of the linear mixed model analysis. The red dotted line represents the estimated mean volume for each level derived from the linear mixed model

The LMM estimated the effects of various factors on the total injected blood volume of EBP (Table 2). GAD-7 and SSS were excluded from the model due to multicollinearity with PHQ-9. The results of the LMM revealed that a lower target level of EBP (per one level, beta = 0.306, *P* = 0.029) and male sex (beta = 4.347, *P* = 0.024) were significantly associated with an increased injected blood volume. Figure 4 shows violin plots of the injected blood volume stratified by spinal levels. Other variables, including BMI, Bern score, and PHQ-9, were not statistically significant in the final model.

**Table 2:** Estimated fixed effects by linear mixed model (LMM)

Variable	Estimate (beta)	95% Confidence interval	*P* value
Intercept	15.497	-1.583–32.578	0.073
**EBP-related Factors**			
- Target level	0.306	0.032–0.581	0.029*
- Days since symptom onset	0.002	-0.002–0.006	0.377
- Blind EBP	2.152	-1.256–5.559	0.206
- Number of EBP	-0.375	-1.045–0.296	0.236
- Cross-sectional area of spinal canal	0.003	-0.027–0.034	0.826
- EBP level within SLEC extension	1.841	-1.467–5.148	0.257
**Demographic Data**			
- Age	-0.101	-0.242–0.041	0.155
- Sex (male vs. female)	4.347	0.645–8.049	0.024*
- BMI	-0.369	-0.798–0.060	0.088
**Imaging Findings**			
- Bern score	-0.062	-0.529–0.653	0.830
- Dorsal SLEC	-0.572	-4.026–2.882	0.735
**Headache Intensity**			
- Maximum intensity (NRS)	-0.148	-0.928–0.632	0.699
**Impact of Headache**			
- HIT-6	0.057	-0.064–0.178	0.335
**Psychological State**			
- GAD-7**			
- PHQ-9	-0.039	-0.266–0.188	0.728
**Somatic Symptom Burden**			
- WPI	0.125	-0.462–0.712	0.665
- SSS**			

* Statistically significant (*P* < 0.05)

** GAD-7 and SSS were excluded from the LMM due to multicollinearity with PHQ-9

BMI: body mass index, EBP: epidural blood patch, SLEC: spinal longitudinal extradural collection, HIT-6: Headache Impact Test-6, NRS: Numeric Rating Scale, GAD-7: Generalized Anxiety Disorder-7, PHQ-9: Patient Health Questionnaire-9, WPI: Widespread Pain Index, SSS: Symptom Severity Scale

A sensitivity analysis was conducted to assess the robustness of the primary findings. In the univariable LMM (Table S1 in Supplementary Material 1), each independent variable was tested separately, and those with *p* < 0.10 were included in the reduced multivariable model (Table S2 in Supplementary Material 1). No further variable exclusion was required due to multicollinearity. The reduced model confirmed the primary findings, with lower target level (beta = 0.328, *P* = 0.007) and male sex (beta = 2.561, *P* = 0.011) remaining significantly associated with higher tolerable EBP volume. Additionally, number of EBP (beta = -0.804, *P* = 0.001) was also a significant factor in the reduced model.

## Discussion

The major findings of this study are as follows: (1) Lower spinal levels were associated with a higher tolerable EBP volume; (2) male patients had an approximate EBP volume that was 3–4 mL greater than that of female patients; and (3) psychological state did not have a significant effect on the tolerable EBP volume.

EBP is indicated for both SIH and post-dural puncture headache (PDPH). In cases of PDPH, EBP blood volumes greater than 20 mL have not demonstrated additional benefits [19]. Conversely, in SIH, recent studies highlight the critical importance of higher injection volumes for treatment success. For instance, Wu et al. reported that among 150 SIH patients, the response rate to a first targeted EBP was 68% when the injected volume exceeded 22.5 mL, compared to 47% for smaller volumes [3]. Similarly, Pagani-Estévez et al. found that in a cohort of 202 SIH patients, higher volumes (> 20 mL) were the only significant factor in multivariable regression (odds ratio 1.64, *p* < 0.0001) [4]. D’Antona et al.‘s meta-analysis also demonstrated response rates of 77% for volumes greater than 20 mL and 66% for volumes of 20 mL or less [6]. These differences between PDPH and SIH likely arise from variations in the location of dural tears and CSF leaks [11].

Our findings contribute to this understanding by emphasising the trade-off between spinal level and tolerable EBP volume. While blind EBP procedures are generally performed at lumbar levels due to their lower risk of complications, achieving the desired EBP volume at cervical or higher thoracic levels may be more challenging due to patient tolerability. This has significant implications for the development of targeted EBP strategies. Previous studies have shown that targeted EBP procedures are more effective than blind EBP procedures. However, cervical or upper thoracic target levels may limit achievable EBP volumes, raising questions about whether a uniform target volume is appropriate across all levels or individuals.

The cross-sectional area of the spinal canal varies significantly by spinal level and among individuals, which may influence the efficacy of EBP [20–22]. Martin et al. suggested that epidural pressure, rather than absolute injection volume, might be more critical determinant of EBP success [5]. In our study, cross-sectional area was significantly associated with tolerable EBP volume in the univariable analysis; however, this association was no longer significant after adjusting for spinal level and sex in the multivariable model.

Nevertheless, male patients tolerated approximately 3–4 mL more EBP volume at the same level compared to female patients. Lin et al. previously reported that female patients had higher EBP success rates with the same injected volume, likely due to their smaller spinal canals, which could lead to increased epidural pressure and more effective sealing of dural tears or CSF leaks [23]. Given these findings, future research should explore whether epidural pressure, calculated as the ratio of injected EBP volume to cross-sectional area, could serve as a predictive marker of EBP success.

In this study, psychological factors did not significantly affect tolerable EBP volume. Since the tolerability of EBP is based on the patient’s subjectiveness, we expected that factors related to pain susceptibility, such as anxiety and somatic symptom burden, would be associated with lower tolerable volumes; however, no such association was observed.

In contrast, the reduced multivariable model revealed a significant trend in which a higher number of EBP procedures was associated with lower tolerable EBP volumes. Rather than suggesting that repeated EBPs lead to reduced tolerance, we interpret this finding as reflecting baseline differences in tolerability among patients. Patients who initially tolerated lower EBP volumes may have been more resistant to EBP procedures overall, leading to longer disease durations and an increased likelihood of undergoing multiple EBP sessions—a potential source of bias in this measurement [24].

This study has several strengths. To our knowledge, it is the first to analyse the factors influencing tolerable EBP volume, incorporating structural, clinical, and psychological elements. Moreover, the standardised EBP protocol in our clinic (single-level, interlaminar approach) minimised confounding variables. Consecutive EBP procedures were performed at least 2 weeks apart, ensuring that residual blood from previous injections did not affect subsequent procedures. Additionally, data from various spinal levels were included, thereby enhancing the robustness of the analysis.

Nevertheless, there are limitations. First, the retrospective design and relatively small sample size may have limited its statistical power. Second, performance or response bias may have influenced the results, as both clinicians and patients may have been more willing to tolerate higher injection volumes at lumbar levels due to the lower perceived risk of complications in this region. Third, our EBP protocol was originally designed for PDPH, restricting maximum injection volumes to 20 mL, which complicates comparisons with studies employing larger-volume EBPs. Some experts have recommended limiting the initial EBP volume to under 20 mL in SIH to minimize the risk of complications [11]; however, this recommendation is primarily based on expert opinion rather than clinical evidence, and the optimal initial volume remains uncertain. Fourth, as this study was conducted at a tertiary center—the only institution in Korea performing DSM—the generalizability of our findings may be limited. This referral bias may have contributed to the relatively high number of total EBP procedures per patient, as our cohort likely included patients who were resistant to prior EBP treatments. However, we assume that this bias had minimal impact on the primary outcome of this study. Fifth, this study did not include an analysis of EBP success rates due to the heterogeneity in the collected assessment of response. Future studies are expected to provide additional clinical insights on EBP response using standardised criteria. Sixth, spinal levels were treated as linear variables in the analysis for simplicity.

## Conclusions

Lower spinal levels and male sex are associated with higher tolerable EBP volumes, while psychological factors, such as anxiety or somatic symptom burden, showed no significant influence. These findings highlight the importance of individual and anatomical factors in optimising EBP strategies for SIH, suggesting that a tailored approach, rather than a fixed volume target, may improve treatment outcomes. The trade-off between spinal level and tolerable volume should be factored into targeted EBP strategies, particularly at cervical or thoracic levels. Future large-scale prospective studies are needed to validate these results and further investigate how structural, clinical, and psychological factors interact to influence EBP success.

## Electronic supplementary material

Below is the link to the electronic supplementary material.

Supplementary Material 1

## Data Availability

No datasets were generated or analysed during the current study.

## Abbreviations

BMI: Body mass index
CSF: Cerebrospinal fluid
CTM: Computed tomography myelography
DSM: Digital subtraction myelography
EBP: Epidural blood patch
GAD-7: Generalized Anxiety Disorder-7
HIT-6: Headache Impact Test-6
IQR: Interquartile range
LMM: Linear mixed model
NRS: Numeric Rating Scale
PDPH: Post-dural puncture headache
PHQ-9: Patient Health Questionnaire-9
SD: Standard deviation
SIH: Spontaneous intracranial hypotension
SLEC: Spinal longitudinal extradural fluid
SSS: Symptom Severity Scale
WPI: Widespread Pain Index
