# Supplementary material for: Lower spinal levels and male sex are associated with greater epidural blood patch volume in spontaneous intracranial hypotension
Source: J Headache Pain. 2025 Apr 14;26(1):75. doi: 10.1186/s10194-025-02015-1 (PMC11995639; doi:10.1186/s10194-025-02015-1)
Supplement: Supplementary file 1 — Supplementary Material 1 [file 10194_2025_2015_MOESM1_ESM.docx]

**Table S1.** Univariable linear mixed model (LMM) analysis of factors associated with injected blood volume

| Variable | Estimate (beta) | 95% Confidence interval | P value |
| --- | --- | --- | --- |
| **EBP-related Factors** |  |  |  |
| - Target level | 0.380 | 0.223 – 0.538 | <0.001* |
| - Days since symptom onset | -0.001 | -0.005 – 0.003 | 0.578 |
| - Blind EBP | 3.611 | 1.555 – 5.668 | 0.001* |
| - Number of EBP | -0.818 | -1.352 – -0.285 | 0.003* |
| - Cross-sectional area of spinal canal | 0.026 | 0.008 – 0.044 | 0.006* |
| - EBP level within SLEC extension | -2.471 | -4.602 – -0.340 | 0.024* |
| **Demographic Data** |  |  |  |
| - Age | 0.044 | -0.061 – 0.149 | 0.408 |
| - Sex (male vs. female) | 3.307 | 1.119 – 5.496 | 0.004* |
| - BMI | 0.047 | -0.240 – 0.335 | 0.742 |
| **Imaging Findings** |  |  |  |
| - Bern score | -0.172 | -0.656 – 0.312 | 0.479 |
| - Dorsal SLEC | 0.523 | -1.887 – 2.934 | 0.665 |
| **Headache Intensity** |  |  |  |
| - Maximum intensity (NRS) | -0.402 | -0.994 – 0.189 | 0.178 |
| **Impact of Headache** |  |  |  |
| - HIT-6 | -0.025 | -0.127 – 0.078 | 0.632 |
| **Psychological State** |  |  |  |
| - GAD-7** | -0.013 | -0.210 – 0.183 | 0.892 |
| - PHQ-9 | -0.064 | -0.233 – 0.104 | 0.446 |
| **Somatic Symptom Burden** |  |  |  |
| - WPI | -0.224 | -0.751 – 0.303 | 0.397 |
| - SSS** | -0.033 | -0.466 – 0.401 | 0.880 |

* Variables with P-value < 0.10 were considered for multivariable analysis.

**Table S2.** Multivariable linear mixed model (LMM) analysis of factors associated with injected blood volume

| Variable | Estimate (beta) | 95% Confidence interval | P value |
| --- | --- | --- | --- |
| Intercept | 8.378 | 3.640 – 13.116 | 0.001 |
| - Target level | 0.3285 | 0.082 – 0.489 | 0.007* |
| - Blind EBP | 1.198 | -1.387 – 3.782 | 0.359 |
| - Number of EBP | -0.804 | -1.277 – -0.330 | 0.001* |
| - Cross-sectional area of spinal canal | 0.007 | -0.013 – 0.027 | 0.497 |
| - EBP level within SLEC extension | 1.133 | -1.427 – 3.692 | 0.381 |
| - Sex (male vs. female) | 2.561 | 0.612 – 4.509 | 0.011* |

Statistically significant variables (P < 0.05) are marked with an asterisk (*).
